# Accelerated versus Standard Corneal Cross-linking for Progressive Keratoconus in Syria

**DOI:** 10.18502/jovr.v16i3.9430

**Published:** 2021-07-29

**Authors:** Abdelrahman M Salman, Taym R Darwish, Yusra H Haddad, Rafea H Shabaan, Mohammad Z Askar

**Affiliations:** ^1^Department of Ophthalmology, Tishreen University, Latakia, Syria; ^2^Department of Ophthalmology, Damascus University, Damascus, Syria; ^3^Tartous University, Faculty of Medicine, Tartous, Syria

**Keywords:** Accelerated, Corneal Crosslinking, HOAs, Keratometry, Posterior Astigmatism, Standard

## Abstract

**Purpose:**

To compare the outcomes of accelerated versus standard corneal cross-linking for the treatment of progressive keratoconus.

**Methods:**

In this retrospective comparative study, 63 eyes of 40 patients with progressive keratoconus were divided into two groups; 27 eyes in group one were treated with an accelerated protocol (10 mW/cm ${}^{2}$ , 9 min) and 36 eyes in group two were treated with the standard method (3 mW/cm ${}^{2}$ , 30 min). Visual acuity, refraction, corneal topography, corneal tomography, and anterior and posterior corneal higher-order aberrations (HOAs) were assessed preoperatively and 18–30 months postoperatively.

**Results:**

The LogMAR uncorrected and corrected distance visual acuity values were improved in both groups postoperatively. However, the improvement was significantly higher in group one (*P* < 0.05, all). The flattening in the anterior keratometry readings, flat K, steep K, and average K were significantly higher in group two (*P* < 0.001, all). The maximum anterior keratometry (AKf) values significantly decreased in both groups, whereas the maximum posterior keratometry (AKb) values increased. The reduction in the minimum corneal thickness (ThKmin) was significantly greater (36.49um) in group two, compared to 10.85um in group one. There was a significant increase in the posterior average keratometry, and a significant decrease in the posterior astigmatism, along 3 mm meridian in S-CXL (*P* = 0.03, *P* = 0.008, respectively), while the corresponding values showed no statistical significance in group one (*P* > 0.05). The anterior corneal trefoil was significantly reduced in group one (*P* = 0.002), whereas anterior total HOAs and coma were significantly improved in group two (*P* < 0.0014, all). The posterior corneal spherical aberration decreased significantly in group one (*P* = 0.02), while group two revealed significant reduction in the posterior trefoil values (*P* = 0.011). The change in the anterior maximum keratometry was significantly and positively correlated to the preoperative maximum keratometry in group two (*P* = 0.53, *P* = 0.003).

**Conclusion:**

An accelerated cross-linking protocol using 10 mW/cm ${}^{2}$ for 9 min showed more visual improvement and less pachymetric reduction when compared to the standard protocol, however, anterior corneal flattening, posterior corneal steepening, and the change in the posterior astigmatism were significantly higher in the standard protocol; while corneal HOAs were improved in both protocols.

##  INTRODUCTION

Corneal cross-linking was first introduced by Wollensask et al in 2003.^[1]^ Zhang et al demonstrated that the term “collagen cross-linking” is misleading, as the actual cross-links, induced by interaction of ultraviolet A (UVA) and riboflavin, occurs between the amino terminals of the collagen side chains and the proteoglycans of the extracellular matrix and not between and within the collagen fibers.^[2]^ The standard corneal cross-linking (S-CXL) reported by Wollensask et al depends on using 3 mW/cm ${}^{2}$ fluence for 30 min to achieve a total irradiance of 5.4 J/cm ${}^{2}$ .^[1]^ More recently, a variety of CXL protocols – in term of fluency, time, epithelial integrity, and different indications for CXL – have been introduced.^[3,4,5]^ The accelerated corneal cross-linking (A-CXL) uses high energy (up to 30 mW/cm ${}^{2}$ ) for shorter time (3–10 min). Bunsen-Rosoe law of reciprocity states that an increased intensity coupled with reduced exposure time theoretically delivers a total dose to the tissue equivalent to that applied in standard treatment.^[6]^ Evaluations of the difference of the outcomes between S-CXL and A-CXL and their impact on the anterior corneal flattening, hyperopic shift, astigmatism, and corneal thinning have gained a particular importance for the refractive surgeons in choosing the CXL protocol to combine with other refractive surgery procedure.^[7,8,9]^

In this study, we aimed to compare the visual outcomes, topographic parameters, and corneal higher-order aberrations (HOAs) values (anterior and posterior) of an A-CXL 10 mW/cm ${}^{2}$ , 9 min and the standard CXL protocol using 3 mW/cm ${}^{2}$ , 30 min.

##  METHODS

This study was approved by the Research Ethics Committee of Tishreen University in accordance with the tenets of the Declaration of Helsinki. Informed consent, in Arabic language, was obtained from all patients over 18 years or their guardians if under 18 at the time of cross-linking. A retrospective, comparative study was performed at the Department of Ophthalmology, Tishreen University Hospital, Syria. All patients who underwent epithelium-off corneal cross-linking between January 2016 and February 2017 were recruited. Sixty-three eyes of 40 patients were included in this analysis. Patients in whom bilateral cross-linking was performed and underwent different types of procedure (A-CXL vs S-CXL) were included to avoid inter-eye correlation. Subjects were divided into two groups: 27 eyes in group one were treated with A-CXL and 36 eyes of group two underwent S-CXL. Diagnosis of keratoconus was made if (a) there was irregular cornea determined by distorted keratometry mires or distortion of the dilated retinoscopic reflex (or combination of these) in addition to (b) at least two of the following topographic/tomographic findings: abnormal posterior ectasia, abnormal thickness distribution, or symmetry index front (SIf) > 1.17 D; or one of the following slit-lamp findings: Vogt striae, 2-mm arc of Fleisher ring, or corneal scaring consistent with keratoconus.^[10]^ Progression of keratoconus was defined as: at least 1 diopter increase in the anterior maximum keratometry (AKf) or in the manifest refraction spherical equivalent (MRSE), decrease of 5% in the minimum pachymetry, or loss of at least two lines of the corrected distance visual acuity (CDVA) during the past 12 months. Patients under 18 years were cross-linked without waiting for progression.^[11]^ Patients who had preoperative pachymetry < 400 µm, previous ocular surgery, corneal scar, pregnancy, lactation, herpetic keratitis, or dry eye were excluded. All patients had comprehensive ocular examination, including the measurement of uncorrected distance visual acuity (UDVA) and CDVA, manifest refraction, slit lamp examination, and funduscopy. Topographic and tomographic measurement, as well as corneal total HOAs, coma, trefoil, and spherical aberrations (anterior and posterior at 6 mm optical zone) were obtained by Placido Scheimpflug-tomographer, Sirius (CSO, Italy). These investigations were carried out in all patients preoperatively and 18–30 months postoperatively. Visual acuity was converted to LogMAR units. All patients were instructed to discontinue the use of hard or soft contact lenses, for at least three and one weeks, respectively, prior to their examination and CXL.

### Surgical Technique

The “epi-off” CXL technique was used in both groups. Topical proparacaine hydrochloride 0.5% (Proparacaine Rama, Rama Pharma, Syria) anesthetic eye drops were administrated every 3 min starting 10–15 min before surgery. The central corneal epithelium (8–9 mm) was removed using blunt spatula and dry sponge, without alcohol assistance. Riboflavin with dextran (0.1% riboflavin in 20% dextran, Medicross, Germany) solution was instilled every 3 min for 60 min, starting 30 min before irradiance and continuing for 30 min during the irradiance, in S-CXL group. In A-CXL group, riboflavin was instilled every 2 min for 29 min, starting 20 min before irradiance and continuing for 9 min during the UVA irradiance. The irradiance was commenced after saturation of the anterior chamber with riboflavin. This was inspected by slit-lamp examination as fluorescence within the anterior chamber. In S-CXL group, eyes were treated with UV-X (Peschke Meditrade Gmbh, Hueneberg, Switzerland) system; 3 mW/cm ${}^{2}$ was applied for 30 min to achieve the total energy of 5.4 J/cm ${}^{2}$ . Eyes of the A-CXL group were irradiated with the Vega C.B.M-X Linker (CSO, Italy) using the A-CXL 10 mW/cm ${}^{2}$ for 9 min to reach the same total energy. After UVA irradiance, the corneal surface was irrigated with balanced salt solution and soft contact lens was applied for 3–4 days. Topical moxifloxacin 0.5% (Megamox, Rama Pharma, Syria) eye drop was prescribed four times daily for one week and topical fluorometholone 0.1% (Methouflor 0.1%, Diamond Pharma, Syria) eye drop was applied four times daily for two weeks, which was then tapered to twice daily for two weeks.

### Statistical Analysis

Statistical Package for Social Sciences Software version 20 (SPSS, INC, Chicago, IL, USA) was applied. Data were expressed as mean ± standard deviation (SD). Two samples independent *T*-test and paired *T*-test were applied for normally distributed variables, while non-parametric test was used if a paired *T*-test was applied to compare the postoperative with the baseline outcomes. *P*-value < 0.05 was considered significant.

##  RESULTS

### Baseline Characteristics

Sixty-three eyes of 40 patients were included in this study. Of these, 27 eyes of 15 patients (9 females, 18 males, mean age; 23.13 ± 7.72 years) underwent A-CXL and 36 eyes of 25 patients (24 females, 12 males, mean age; 23.4 ± 7.37 years) underwent S-CXL. There was no significant difference between the two groups in terms of demographic, UDVA, CDVA, MRSE, topography, pachymetry, and corneal HOAs except for anterior trefoil values, 0.94 ± 48 µm in A-CXL group versus 0.64 ± 0.41 µm in S-CXL (*P* = 0.011). Females were significantly higher in A-CXL group (*P* = 0.009) [Tables 1 and 4].

**Table 1:** Patient characteristics at baseline


	**Treatment type**		
	**Tech 9 min**	**Tech 30 min**	
*N* (%)			
No. of subjects	15 (37.5)	25 (62.5)	
No. of eyes	27 (42.86)	36 (57.14)	
		**Total**	***P*** **-value**
Sex, *n* (%)	M	18 (66.67)	12 (33.33)	30 (47.62)	**0.009**
	F	9 (33.33)	24 (67.67)	33 (52.38)	
Eye, *n* (%)	OD	16 (59.26)	19 (52.78)	35 (55.56)	0.608
	OS	11 (40.74)	17 (47.22)	28 (44.44)	
Age, mean ± SD	23.13 ± 7.72	23.4 ± 7.37	23.3 ± 7.4	0.9138
P, paired test; Values in bold are significant (*P* < 0.05)					

**Table 2:** Visual, refractive, and anterior keratometric outcomes


	**Tech 9 min (** ***n*** ** = 27)**		**Tech 30 min (** ***n*** ** = 36)**		
	**Mean**	**Std. Dev.**	**Mean**	**Std. Dev.**	***P*** **-value**
UDVA (LogMAR)	Preoperative	0.66	0.46	0.57	0.37	0.3433
	Postoperative	0.52	0.41	0.50	0.36	0.8176
	Mean change	0.14	0.20	0.07	0.26	0.2825
	P* value	**0.0029**	0.11	
CDVA (LogMAR)	Preoperative	0.32	0.27	0.32	0.23	0.8333
	Postoperative	0.27	0.21	0.29	0.23	0.7371
	Mean change	0.06	0.11	0.03	0.20	0.5167
	P* value	**0.03**	0.37	
MRSE (D)	Preoperative	–2.49	2.89	–1.76	1.04	0.1623
	Postoperative	–2.78	3.65	–1.29	1.71	**0.042**
	Mean change	0.28	2.99	–0.47	1.33	0.2004
	P* value	0.65	**0.05**	
Topo Cyl (D)	Preoperative	–3.24	1.81	–2.83	1.39	0.2531
	Postoperative	–3.10	1.39	–2.61	1.25	0.1716
	Mean change	–0.14	0.77	–0.22	0.71	0.695
	P* value	0.39	0.08	
K1 (D)	Preoperative	44.91	0.34	45.50	3.08	0.3498
	Postoperative	44.73	0.35	44.03	2.57	0.2426
	Mean change	0.19	0.14	1.47	1.54	**0.0001**
	P* value	0.20	** < 0.0001**	
K2 (D)	Preoperative	48.44	2.38	48.21	3.12	0.8272
	Postoperative	48.55	3.06	47.00	2.77	**0.0431**
	Mean change	–0.11	1.39	1.21	1.20	**0.0002**
	P* value	0.69	** < 0.0001**	
Avg K (D)	Preoperative	46.83	2.08	46.80	3.01	0.9575
	Postoperative	46.86	2.61	45.46	2.55	**0.0403**
	Mean change	–0.04	1.99	1.34	1.31	**0.0019**
	P* value	0.9211	** < 0.0001**	
AKf (D)	Preoperative	55.04	4.09	54.78	3.84	0.7238
	Postoperative	54.24	4.61	52.84	3.31	0.0531
	Mean change	0.80	1.39	1.93	2.73	0.1205
	P* value	**0.0312**	**0.0011**	
UDVA, uncorrected distance visual acuity; CDVA, corrected distance visual acuity; MRSE, manifest refraction spherical equivalent; Cyl, sim cylinder value; K1, flat keratometry; K2, steep keratometry; AvgK, anterior average keratometry; AKf, apical keratoscopy front; D, diopter P, Paired test; P*, student's test; Values in bold are significant (*P* < 0.05)						

**Table 3:** Pachymetry, posterior corneal keratometries, and astigmatism outcomes


	**Tech 9 min (** ***n*** ** = 27)**		**Tech 30 min (** ***n*** ** = 36)**		
	**Mean**	**Std. Dev.**	**Mean**	**Std. Dev.**	***P*** **-value**
ThkMin (µm)	Preoperative	436.54	26.51	449.46	33.81	0.1062
	Postoperative	425.69	27.55	412.97	47.23	0.2252
	Mean change	10.85	22.05	36.49	51.07	**0.0198**
	P* value	**0.02**	**0.0002**	
AKb (D)	Preoperative	77.08	7.69	78.69	8.74	0.9647
	Postoperative	79.25	8.33	81.08	9.56	0.6893
	Mean change	–2.17	4.01	–2.39	9.73	0.93
	P* value	**0.0406**	0.2315	
AvgK (bck, 3) (D)	Preoperative	–7.22	0.70	–7.03	0.86	0.5818
	Postoperative	–7.20	0.94	–6.66	1.29	0.0731
	Mean change	–0.02	0.66	–0.36	0.97	0.1276
	P* value	0.9131	**0.0386**	
AvgK (bck, 5) (D)	Preoperative	–6.97	0.48	–6.88	0.74	0.787
	Postoperative	–6.97	0.61	–6.59	0.93	0.0637
	Mean change	0.00	0.42	–0.29	0.60	**0.0436**
	P* value	0.9609	**0.011**	
Cyl (back, 3) (D)	Preoperative	–0.92	1.20	–1.09	0.53	0.3278
	Postoperative	–1.36	0.74	–2.19	2.12	0.1088
	Mean change	0.44	1.25	1.10	2.18	0.186
	P* value	0.1045	**0.0086**	
Cyl (back, 5) (D)	Preoperative	–0.71	0.82	–0.88	0.55	0.7552
	Postoperative	–1.21	1.22	–1.79	2.11	0.2665
	Mean change	0.50	1.32	0.91	1.85	0.3643
	P* value	0.0819	**0.0092**	
ThiKMin, minimum corneal thickness; AKb, apical kertoscopy back; AvgK (bck, 3), average keratometry along 3 mm back meridian; AvgK (bck, 5), average keratometry along 5 mm back meridian; Cyl (bck, 3), cylinder value along 3 mm back meridian; Cyl (bck, 5), cylinder value along 5 mm back meridian; D, diopter P, Paired test; P*, student's test; Values in bold are significant (*P* < 0.05)						

**Table 4:** Anterior and posterior corneal HOAS outcomes


	**Tech 9 min (** ***n*** ** = 27)**		**Tech 30 min (** ***n*** ** = 36)**		
	**Mean**	**Std. Dev.**	**Mean**	**Std. Dev.**	***P*** **-value**
Anterior corneal HOAs 6 mm						
Anterior total HOAS (RMS, µm)	Preoperative	2.35	1.27	2.62	1.30	0.4248
	Postoperative	2.25	1.27	2.16	1.13	0.5737
	Mean change	0.10	0.33	0.46	0.73	**0.0269**
	P* value	0.1329	**0.0008**	
Trefoil (RMS, µm)	Preoperative	0.94	0.48	0.64	0.41	**0.0115**
	Postoperative	0.82	0.50	0.72	0.40	0.399
	Mean change	0.12	0.18	–0.08	0.42	**0.0269**
	P* value	**0.0024**	0.2688	
Coma (RMS, µm)	Preoperative	2.04	1.17	2.26	1.31	0.5091
	Postoperative	1.94	1.32	1.82	1.13	0.5074
	Mean change	0.10	0.41	0.44	0.74	**0.0433**
	P* value	0.2323	**0.0013**	
Spherical aberration (RMS, µm)	Preoperative	0.11	0.37	0.05	0.45	0.5833
	Postoperative	0.08	0.34	0.01	0.38	0.3639
	Mean change	0.04	0.15	0.04	0.31	0.9096
	P* value	0.2567	0.4272	
Posterior corneal HOAs 6 mm						
Posterior total HOAS (RMS, µm)	Preoperative	0.86	0.49	1.19	1.00	0.0923
	Postoperative	0.93	0.50	0.90	0.57	0.8215
	Mean change	–0.07	0.29	0.28	0.84	**0.037**
	P* value	0.1912	0.0636	
Trefoil (RMS, µm)	Preoperative	0.56	0.43	0.69	0.61	0.3604
	Postoperative	0.62	0.44	0.46	0.33	0.106
	Mean change	–0.05	0.27	0.23	0.52	**0.0115**
	P* value	0.2944	**0.0146**	
Coma (RMS, µm)	Preoperative	0.38	0.21	0.54	0.45	0.052
	Postoperative	0.38	0.20	0.44	0.26	0.3425
	Mean change	0.00	0.12	0.10	0.40	0.2003
	P* value	0.8433	0.1594	
Spherical aberration (RMS, µm)	Preoperative	–0.05	0.09	–0.14	0.27	0.209
	Postoperative	–0.08	0.07	–0.14	0.22	0.106
	Mean change	0.03	0.07	0.00	0.24	0.519
	P* value	**0.027**	0.9096	
HOAs, higher order aberrations; RMS, root mean square P, Paired test; P*, student's test; Values in bold are significant (*P* < 0.05)						

**Table 5:** Correlation between AKf Change at follow-up and preoperative assessment variables


**AKf change**	**Preop UDVA**	**Preop CDVA**	**Preop ThiKMin**	**Preop AKf**
Tech 9 min	R	0.3855	0.1228	–0.4321	–0.2302
	*P*-value	0.1265	0.6387	0.0832	0.374
Tech 30 min	R	–0.0207	0.0845	–0.0711	0.5365
	*P*-value	0.9182	0.6751	0.7246	**0.0039**
UDVA, uncorrected distance visual acuity; CDVA, corrected distance visual acuity; ThiKMin, minimum corneal thickness; AKf, apical keratoscopy front P, Student's test; Values in bold are significant (*P* < 0.05); R, correlation coefficient					

### Visual, Refractive, and Anterior Keratometry Outcomes

The UDVA and CDVA values were significantly improved postoperatively compared with baseline in A-CXL group (*P* < 0.05). In S-CXL, the UDVA and CDVA values were improved but the improvement did not reach statistical significance (*P* > 0.05). In terms of mean MRSE, there was a nonsignificant myopic shift in group one, and significant hyperopic shift in group two (A-CXL: 0.28 D, *P* = 0.65; S-CXL: –0.47 D, *P* = 0.05). Both groups had slight reduction in the Sim cylinder values postoperatively (A-CXL: –0.14 D, *P* = 0.39, S-CXL: –0.22 D, *P* = 0.08). K1, K2, and Avg K did not show significant change in A-CXL group (0.19 D, –0.11 D, –0.04 D, respectively) (*P* ≥ 0.2, all), while the corresponding values were significantly decreased in S-CXL group (K1: 1.47 D, K2: 1.21 D, Avg K: 1.34 D, *P* < 0.001, all). AKf (maximum anterior keratometry) values were significantly reduced in both groups (A-CXL; 0.8 D, *P* = 0.03, S-CXL; 1.93 D, *P* = 0.001) [Table 2].

**Figure 1:**
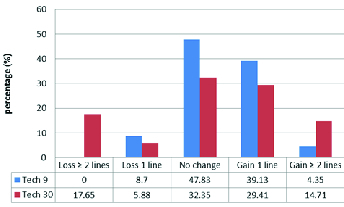
corrected distance visual acuity changes after 9 and 30 min corneal cross-linking (Snellen).

The change in Snellen CDVA in both groups was as follows: in the A-CXL group, 39.13% and in the S-CXL group, 29.41% gained one line postoperatively. The corresponding values were 4.35% and 14.71% regarding the gain of two or more lines, respectively. In the A-CXL group, 8.7% and in the S-CXL group, 5.88% lost one line. The corresponding figures were 0.0% and 17.65% in terms of the loss of two or more lines, respectively. Totally, 47.83% of eyes in the A-CXL group and 32.35% in the S-CXL group displayed no change [Figure 1].

### Pachymetry, Posterior Corneal Keratometry, and Astigmatism Outcomes

The minimum corneal thickness was reduced 10.85 µm in the A-CXL group and 36.49 µm in the S-CXL group (*P* = 0.029 and *P* = 0.002, respectively). The baseline values of AKb (the steepest point of the posterior surface) increased from 77.08 D to 79.25 D in the A-CXL group, and from 78.08 D to 80.08 D in the S-CXL group. The average posterior keratometry Avg K(bck) along 3- and 5-mm back meridians were not significantly changed in the A-CXL group (*P* > 0.9, all), while they significantly increased (representing posterior steepening) in the S-CXL group (*P* < 0.05, all). The posterior corneal astigmatism (PCA) did not significantly change along the corresponding meridians in the A-CXL group (*P* > 0.5, all). In the S-CXL group, PCA values decreased (absolute values increased) significantly in the 3- and 5-mm meridians (*P* < 0.009, all) [Table 3].

### Anterior and Posterior Corneal Higher-order Aberrations Outcomes

In the A-CXL group, the anterior trefoil was significantly decreased (*P* = 0.0024), whereas total HOAs, coma, and spherical aberrations showed no statistically significant difference (*P* > 0.05, all). The anterior total HOAs and coma aberrations were significantly improved in the S-CXL group (*P* = 0.0008 and *P* = 0.001, respectively), while the trefoil and the spherical aberrations revealed nonsignificant change (*P* > 0.02, all). There was a significant reduction in the posterior spherical aberration values (*P* = 0.0027) in the A-CXL group; while the posterior total HOAs, trefoil, and coma did not significantly change (*P* > 0.01, all). The total HOAs, coma, and spherical aberrations were not significantly changed in the S-CXL group (*P* > 0.014, all), while the trefoil significantly decreased (*P* = 0.014) [Table 4].

### Correlation Analysis Outcomes

Both groups revealed no significant correlation between the preoperative measurements (UDVA, CDVA, and ThKMin) and the change in AKf values at the follow-up. However, the change in AKf was significantly and positively correlated to the preoperative AKf in group two (R = 0.53, *P* = 0.003) [Table 5].

The mean difference for each parameter from baseline and final follow-up time were compared in both groups. The changes were not statistically significant between the two groups, except for ThKmin, Sim keratometry (K1, K2, KAvg), KAvg (bck, 5), anterior total HOAs, trefoil and coma, and posterior total HOAs and trefoil (*P* < 0.05, for all) [Tables 2–4].

##  DISCUSSION

Several studies have revealed that both A-CXL and S-CXL are effective in halting the progression of keratoconus.^[12,13]^ Tomita et al published the first article comparing the standard and accelerated protocols.^[14]^ They reported no significant difference in the mean UDVA and CDVA, keratometric readings, or the postoperative MRSE values. In contrast, we found improvement in the logMAR visual acuity in both groups. The UDVA and CDVA significantly improved (0.14, *P* = 0.002 and 0.06, *P* = 0.03, respectively) in the A-CXL group, compared to 0.07, *P* = 0.11 and 0.03, *P* = 0.37, respectively, in the S-CXL group. There was no statistically significant difference between the two groups. However, 12 eyes of the A-CXL group lost two lines of the Snellen DCVA at three month postoperatively. The reduction in visual acuity at this stage was attributed to the increase in the corneal HOAs and the decrease in the contrast sensitivity. Ghanavati et al stated that increased corneal HOAs and decreased contrast sensitivity were the factors responsible for deceased visual acuity at the early postoperative period after cross-linking.^[15]^ Fortunately, none of the eyes treated with A-CXL lost two or more lines of Snellen CDVA at the final follow-up. However, this was not the case for the eyes treated with S-CXL, as six eyes lost two or more lines at the final examination. This could be explained by the significant persistent haze formation in four eyes and scar development in two eyes. Our study showed a slight myopic shift of the MRSE in the A-CXL group and significant hyperopic shift (O.47 D) in the S-CXL group, while Sim cylinder values were nonsignificantly reduced in both groups. Hashemi et al reported a significant reduction in keratometric readings in the S-CXL treated eyes but not in the A-CXL treated ones and concluded that the flattening effect was higher in the S-CXL group.^[16]^ This is in agreement with our results. We found significant flattening in the mean flat, steep, and average keratometry values (1.47 D, 1.20 D, 1.99 D, respectively) with the S-CXL protocol, while the corresponding values did not show any statistically significantly deference in the A-CXL group. The anterior maximum keratometry or K max, which is considered as the most sensitive indicator for KC progression,^[17,18]^ was significantly reduced after treatment in both A-CXL and S-CXL groups (*P* < 0.05, all). The mean ThKmin values decreased 10.85 µm in the A-CXL group compared to 36.49 µm in the S-CXL group. Shetty et al observed that the minimum thickness reduction was higher in the S-CXL group.^[19]^ Greenstein et al suggested that the decrease in thickness was related to an increased compactness of the cross-linked cornea.^[20]^ However, the marked reduction in corneal thickness after S-CXL in our study may represent a measurement artefact. Dependence on Scheimpfug-based pachymetric measurements was one of our study limitations. Anterior segment optical coherence tomography (AS-OCT) showed higher repeatability compared with Scheimpflug imaging devices in measuring the corneal thickness.^[21]^

Patients with KC are more likely to develop cataract at a younger age than normal subjects^[22]^ and many of them will eventually require cataract surgery and toric intraocular lens implantation. Koch et al^[23]^ reported that neglecting PCA which is higher in KC patients compared to normal population^[24]^ will lead to the overcorrection in eyes having with the rule astigmatism and the undercorrection in eyes with the against the rule astigmatism. Safarzadeh et al found a nonsignificant difference in PCA and a significant increase in the mean posterior maximum keratometry values after S-CXL treatment.^[25]^ In contrast, we evaluated the changes of posterior astigmatism and posterior keratometry along the 3- and 5-mm meridians between the baseline and the postoperative follow-up in both groups. The Cyl (bck) values decreased (absolute values increased) nonsignificantly along with the studied meridians in the A-CXL group. While the decrease was significant in the S-CXL group. The difference in Cyl (bck) values along the 3-mm meridian was 0.44 D in the A-CXL (*P* = 0.1) and 1.10 D in the S-CXL (*P* = 0.0086) groups. The mean posterior keratometry AvgK (bck3) increased (absolute values decreased) nonsignificantly in the A-CXL group, while the increase was significant in the S-CXL group along the 3-mm meridian (0.02 D, *P* = 0.91; 0.36 D, *P* = 0.038, respectively). AKb values (the steepest point of the posterior surface) increased postoperatively, 2.17 D in the A-CXL group and 2.39 D in the S-CXL group. We hypothesize the difference in PCA outcomes between our study and the study by Safarzadeh et al can be attributed to the difference in the type of the topographer used, as Safarzadeh depended on Pentacam measurement. Although Pentacam and Sirius are both Scheimpfug-based tomographers, Sirius showed good to excellent repeatability^[26,27,28]^ and was less affected than Pentacam by the post-CXL haze.^[29]^ The increase in both the posterior mean and maximum keratometries represent increased posterior corneal steepening. Twa et al evaluated the corneal changes after 5,211 myopic LASIK procedures. They suggested that the posterior steepening is a response to the anterior flattening induced by myopic LASIK correction.^[30]^ Kirgiz et al considered posterior corneal steepening as important as anterior corneal flattening for stabilizing the keratometric values and enhancing the visual acuity.^[31]^

Recent studies have revealed increased spherical and coma aberrations in eyes with KC compared to normal population.^[32]^ Greenstein et al reported significant improvement in the anterior corneal HOAs and coma after S-CXL treatment.^[33]^ However, they found no statistically significant difference in the posterior corneal HOAs. This is to some extent in agreement with our findings; anterior total HOAs and coma were significantly reduced (*P* < 0.0014, all), while posterior corneal trefoil was significantly deceased in the S-CXL group. Ozulken et al found significant difference in coma aberrations after 10 min at 9 mW/cm ${}^{2}$ UVA irradiance.^[34]^ In our study, anterior trefoil and posterior spherical aberrations values were significantly improved in the A-CXL group (*P* = 0.002 and *P* = 0.02, respectively). Ghanem et al concluded that the improvement in HOAs in KC patients is attributed to the flattening of the corneal apex caused by the CXL effect.^[35]^

To the best of our knowledge, this the first study to compare the impact of different CXL protocols on the PCA. Lack of demarcation line depth measurements, low number of patients, and the retrospective design of the study were limiting factors in this study. Larger cohort studies to evaluate the effect of CXL on the orientation of the astigmatism and the correlation between the anterior and posterior astigmatism changes are needed.

In summary, we found that S-CXL resulted in significantly higher anterior corneal flattening, more increase in posterior steepening, further decrease in posterior astigmatism, and more reduction in the minimum thickness than the accelerated-CXL. However, both protocols showed improvement in the postoperative visual acuity and the corneal HOAs, but the improvement in the visual acuity was significantly higher in the A-CXL protocol.

##  Financial Support and Sponsorship

Nil.

##  Conflicts of Interest

There are no conflicts of interest.
